# Investigating the Complications and Causes of Failure of the AngioVac System: A Post-Marketing Surveillance From the MAUDE Database

**DOI:** 10.7759/cureus.43720

**Published:** 2023-08-18

**Authors:** Chaitu Dandu, Sardar Muhammad Alamzaib, Dhruvil Patel, Ryan Naughton3, Aws Polina, Maria Najam, Rashid Alhusain, Neel Patel, Yasar Sattar, M. Chadi Alraies

**Affiliations:** 1 Vascular Surgery, Wayne State University School of Medicine, Detroit, USA; 2 Cardiovascular Medicine, Marshall University Joan C. Edwards School of Medicine, Huntington, USA; 3 Internal Medicine, Wayne State University School of Medicine, Detroit, USA; 4 Internal Medicine, Wayne State University Detroit Medical Center, Detroit, USA; 5 Anesthesiology, University of Miami Miller School of Medicine, Jackson Memorial Hospital, Miami, USA; 6 Internal Medicine, Knapp Medical Center, University of Texas Rio Grande Valley, Weslaco, USA; 7 Internal Medicine, Detroit Medical Centre, Detroit, USA; 8 Internal Medicine, New York Medical College/Landmark Medical Center, Woonsocket, USA; 9 Internal Medicine, Icahn School of Medicine at Mount Sinai, New York City, USA; 10 Cardiology, Detroit Medical Centre, Detroit, USA

**Keywords:** thrombectomy, intervention, maude, complications, angiovac system

## Abstract

Background

Aspiration thrombectomy devices, such as the AngioVac, allow the removal of thrombus, especially in patients with contraindications to anticoagulation use. The AngioVac was approved by the U.S. Food and Drug Administration to remove fresh, soft thrombi or emboli during extracorporeal bypass for up to six hours. Real-world data on the most common modes of failure and complications associated with the AngioVac are unavailable.

Methods

The Manufacturer and User Facility Device Experience database was queried for reports of the AngioVac device failure and adverse events from April 2013 to March 2022. Categorical variables were described as numbers, and all statistical calculations were performed with IBM SPSS Statistics, version 27.0 (IBM Corp., Armonk, NY).

Results

A total of 115 events were reported during the study period. After the exclusion of duplicate reports, the final cohort included 93 reports. The most common mode of failure for the AngioVac was physical damage of the device, with 13 reports (14%). The most common vessels associated with events were the superior vena cava and inferior vena cava, occurring in 23 reports (24.7%). The most common adverse clinical events were pulmonary embolism (PE), occurring in 33 reports (35.5%), and perforation, occurring in 16 reports (17.2%). Other less frequent adverse outcomes were arrhythmias, stroke, and foreign body device embedment. There were 45 deaths reported with the use of the AngioVac.

Conclusions

Aspiration thrombectomy devices provide promising efficacy; however, physicians should be aware of known adverse outcomes, even if they are infrequent. Based on this analysis, PE and vessel perforation were the most common adverse outcomes. Furthermore, the most common mode of failure was secondary to physical damage of the device.

## Introduction

The AngioVac system (AngioDynamics, Latham, NY) is a large bore, highly steerable thrombectomy device used for aspiration of thrombi from both intracardiac and vascular locations. Specific applications include the removal of these thrombi from large venous and arterial vessels, valvular vegetation aspiration to enhance surgical preparation, and removal of pulmonary emboli in both sub-acute and acute presentations [1-4]. Unlike other thrombectomy systems, the AngioVac system creates a local suction force at the tip of its cannula to absorb whole clots without using mechanical fragmentation or thrombolytic agents, theoretically reducing the risk of distal embolism [5]. Patient blood is also aspirated in this process, which is filtered for the thrombi and later reintroduced back into the patient’s circulation. The system comes in two pieces: the AngioVac cannula (18F or 22F) and the AngioVac circuit. When the system is ready to be used, the cannula is connected to a bypass pump that establishes a centrifugal suction that facilitates the aspiration of local contents at the edge of the tip 2. The system uses a proprietary expandable funnel mechanism at the tip of the cannula to be introduced proximal to the clot, allowing for debulking and removal with minimal shearing forces [6,7].

As a result, AngioVac is a safer alternative with lower morbidity and mortality than surgical embolectomy and drastically reduces bleeding complication risks when compared with solely thrombolytic therapies. Among high-risk patients, AngioVac elicits minimal circulatory stress that contributes to reduced post-procedural complications, making it a successful alternative to the surgical standard of care [8,9]. Despite its widespread use, limited information on its safety, efficacy, and failure modes is available in a real-world context. 

The purpose of this study was to determine the most common complications and failure modes reported in the Manufacturer and User Facility Device Experience (MAUDE) database, which is an index of failure modes and complications of Food and Drug Administration (FDA)-approved devices [10]. Although single reports have found complications such as ineffective removal, embolism, and device malfunction, there has yet to be published evidence of trends and patterns in these paradigms from real-world settings [11,12]. Herein, we report our results from analyzing these reports over the last decade to characterize trends and patterns to better inform operators of these events.

This article was previously presented as a meeting abstract at the 2022 Transcatheter Cardiovascular Therapeutics (TCT) Conference on September 18, 2022.

## Materials and methods

Data source

The FDA created the MAUDE database, listing adverse events caused by approved medical devices. The MAUDE database contains reports submitted to the FDA by mandatory reporters (manufacturers, importers, and device user facilities) and voluntary reporters, such as healthcare professionals, patients, and consumers. The MAUDE database is publicly available online and de-identified. Therefore, no institutional review board approval was required for this study. We queried the database from April 2013 to March 2022, using the keyword “Angiovac.” 

Outcomes and statistical analysis

The primary outcome of this study was the failure modes of the AngioVac. Secondary outcomes include major complications associated with device failure. Target vessels and their relationship with failure outcomes were also analyzed. The MAUDE database cannot capture the overall utilization of AngioVac in the United States; therefore, the actual incidence rate of each failure or complication type cannot be assessed. Categorical variables were described as numbers, and all statistical calculations were performed with IBM SPSS Statistics, version 27.0 (IBM Corp., Armonk, NY).

## Results

A total of 115 reports were found during the study period. After excluding duplicate reports (n = 21), our final cohort included 93 reports (Table 1). Superior vena cava (SVC) and inferior vena cava (IVC) were the most common target vessels of intervention for clot removal implicated in events (n = 24, 25.8%), followed by the right atrium (n = 19, 20.4%), tricuspid valve (n = 10, 10.78%), right ventricle (n = 7, 7.5%), and pulmonic valve (n = 7, 7.5%).

**Table 1:** Summary of MAUDE reports of the AngioVac device categorized by the paradigms pertaining to clinical utility including the target of device implementation, failure method, clinical consequence, and the patient outcome MAUDE, Manufacturer and User Facility Device Experience

Total number of events	93
Target	
Super vena cava/inferior vena cava, n (%)	24 (25.8%)
Right atrium, n (%)	19 (20.4%)
Tricuspid valve, n (%)	10 (10.8%)
Right ventricle, n (%)	7 (7.5%)
Pulmonic valve, n (%)	7 (7.5%)
Insufficient information, n (%)	18 (19.4%)
Other, n (%)	8 (8.6%)
Failure method	
Pump failure, n (%)	0 (0.0%)
Position failure, n (%)	2 (2.2%)
Air leak/bubbles, n (%)	2 (2.2%)
Occlusion, n (%)	5 (5.4%)
Break/detachment, n (%)	13 (14.0%)
Other, n (%)	8 (8.6%)
None, n (%)	63 (67.7%)
Clinical consequence	93
Perforation, n (%)	16 (17.2%)
Hematoma, n (%)	1 (1.1%)
Foreign body device embedded in tissue, n (%)	0 (0.0%)
Pulmonary embolism, n (%)	34 (36.6%)
Stroke, n (%)	3 (3.2%)
Limb ischemia, n (%)	0 (0.0%)
Arrhythmia, n (%)	4 (4.3%)
Myocardial infarction, n (%)	0 (0.0%)
Other, n (%)	15 (16.1%)
No clinical consequence reported, n (%)	20 (21.5%)
Patient outcome	
Death, n (%)	45 (48.4%)
No consequences, n (%)	10 (10.8%)
Recovered, n (%)	22 (23.7%)
Insufficient information, n (%)	16 (17.2%)

**Figure 1:**
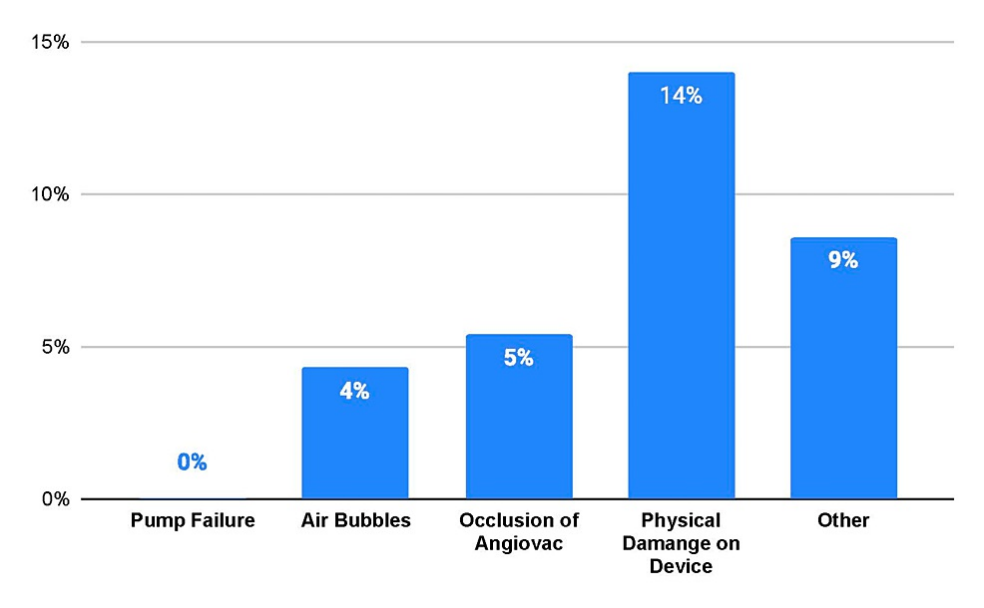
Modes of failure of AngioVac Failure mode categories of the AngioVac device from the MAUDE database reports Physical damage of the device comprised the greatest proportion of reports MAUDE, Manufacturer and User Facility Device Experience

The most common failure mode (Figure 1) was physical damage to the device (n = 13, 14.0%), followed by occlusion of AngioVac (n = 5, 5.4%) and air bubbles (n = 2, 2.2%). In all, 8.6% (n = 8) of the reports stated that the device failed in a way that could not be easily classified in the defined categories. No evidence of pump failure was reported. Physical damage to the device included breaks (n = 2, 2.2%), deflation and inflation problems (n = 3, 3.3%), entrapment of the device (n = 2, 2.2%), failure to advance (n = 3, 3.3%), and insufficient training on device use (n = 3, 3.3%).

**Figure 2:**
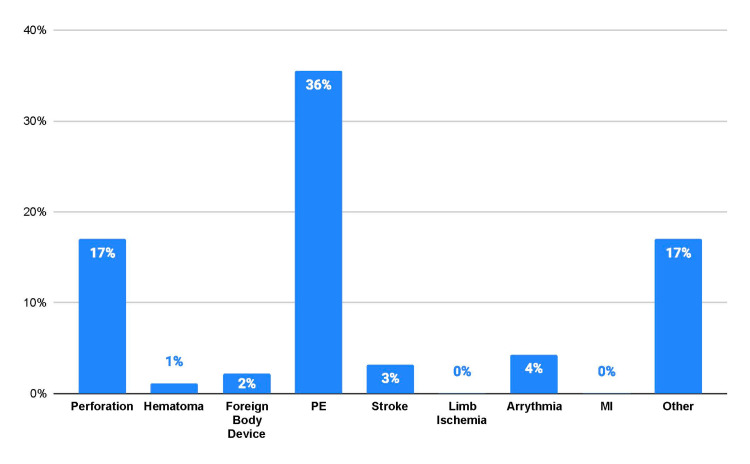
Adverse outcomes The relative proportion of adverse patient outcomes collected in MAUDE reports of the AngioVac device PE comprised the greater proportion of procedural complications PE, pulmonary embolism; MI, myocardial infarction; MAUDE, Manufacturer and User Facility Device Experience

Adverse events associated with AngioVac use were also reported (Figure 2). The most common clinical adverse event was a pulmonary embolism (PE) (n = 34, 36.6%), followed by perforation (n = 16, 17.2%), arrhythmia (n = 4, 4.3%), stroke (n = 3, 3.2%), and hematoma (n = 1, 1.1%). Of the 16 perforation events, two occurred during AngioVac usage in the great vessels, and the remaining 14 occurred during AngioVac usage in the heart. Another 17% of adverse events were reported, but there was no identifiable cause for the adverse event. No foreign body embedment in tissue from the device, myocardial infarction, or limb ischemia complications were reported. Interestingly, 58.1% of adverse events occurred without an identified device or use problems.

## Discussion

Since its FDA approval in March of 2014, the AngioVac cannula has been widely utilized to suction thrombus, emboli, and vegetations in the caval system, right heart system, and pulmonary arteries [13]. Despite the growing use of the AngioVac suction thrombectomy system, no database-wide study has been performed on this device to outline and report common modes of failure and associated clinical outcomes. 

The following are the pertinent findings from our analysis of the MAUDE database reports of the AngioVac device failure modes and complications: (1) PE followed by vessel perforation were the most commonly clinical adverse events associated with the device use, and (2) primary physical damage of the device followed by occlusion of the suctioning lumen were the most common device complications observed in the periprocedural period.

Our study has found that PE was reported in approximately a third of all reported AngioVac device-related complications. Reports have shown that the embolization is likely attributed to fragmentation, particularly during interventions into the right heart and/or caval system that can range from benign effects to severe hemodynamic collapse [14]. Furthermore, materials that are loosely adherent or mobile may also result in a higher likelihood of being embolized during suction thrombectomy with AngioVac. Interestingly, the RAPID registry, which consists of 234 patients from multiple centers who underwent treatment with AngioVac, reported seven cases (3.9%) of distal embolization, of which five were to the pulmonary system [15]. This stands in stark contrast with our data that reported a 33% rate of PEs. Since the MAUDE database does not report underlying patient information, it is possible that these patients may have been more clinically unstable compared to those in the RAPID registry. 

Furthermore, since the MAUDE database is a collection of adverse events associated with the device use, reported events on patient outcomes are more likely to be those that cause significant patient harm or even death compared to less severe clinical complications periprocedurally. Currently, there are no reports or guidelines indicating how to manage PE complications related to AngioVac. Furthermore, using the AngioVac device itself to recover these fragments, while not reported, may be ill-advised as evidence suggests limited efficacy for PE collection in comparison to other regions of the body. This may be attributed to the tortuousness of pulmonary vasculature that may limit device integrity, potentially worsen fragmentation, and consequently embolize regions that are more difficult to intervene in [16]. Despite these concerns regarding PE complications, AngioVac is still considered to be an effective alternative to surgical embolectomy and demonstrates a benefit in treating patients with a primary diagnosis of PE [16,17]. At present, there are no established guidelines or methods for preventing clot fragmentation during AngioVac usage; however, our findings indicate that this complication may frequently occur, and as such, operators must stay alert and ready to address it. 

Perforation of the intervened vessels and heart also comprised a large proportion of AngioVac device-related clinical adverse outcomes in our analysis. The AngioVac device works through suctioning via a catheter lumen that is encased by an umbrella that expands at the site of collection. Current literature and the RAPID registry have reported occurrences of cardiac perforation during AngioVac procedures [15,18]. However, we are the first to report perforation events occurring in the caval system during the procedure. Since the MAUDE database does not mandate recording-specific patient health information and procedural characteristics, it is difficult to ascertain why the perforations occurred in many cases. We hypothesize that causes of perforation can occur during catheter movement, especially in areas with increased tortuousness. As such, it is imperative that the operator remain vigilant and avoid any aggressive maneuvers that may increase the chances of perforation. 

The most common source of device failure was breakage of the device. This includes both the introduction tip and the catheter. While it was unclear if a majority of events occurred due to prior catheter damage present from manufacturing or if the events were due to device operation, operators should confirm that these points of damage are not present prior to device implementation. Secondarily, occlusion of the device from a large thrombus burden or from aspiration of air that produces obstructive bubbles was also a notable source of device failure in our cohort. The presence of air bubbles was a cause for the procedure of abortion. During device initialization, a bypass circuit is formed during priming, which evaluates the presence of air bubbles [5]. Thereby, air bubble introduction is likely a product of aspiration suction being altered during clot evacuation. We recommend that expecting this phenomenon is important for operators as the presence of air bubbles during device operation is likely during times of device interaction with the clot, which can immediately require procedure abortion and evaluation of decision-making for clot release. 

Limitations

Our study is a retrospective analysis of the MAUDE database. The data are collected by voluntary reporting from healthcare professionals when devices fail. As such, this leads to selection bias and overreporting of these adverse events. Additionally, due to limitations of the database, it is difficult to ascertain how much of the clinically adverse events can be directly attributable to devices themselves vs patients who are already at high risk for having adverse events. The data from MAUDE alone cannot be utilized to track trends in adverse event rates for devices over time. Data from reports cannot be extrapolated to determine data about the existence or frequency of problems associated with devices at a population-wide level. Finally, the MAUDE database is a post-marketing surveillance of products; therefore, it can have recall bias or performer bias. Our paper is solely for educational purposes, and it is not a source for any libel/defamation of any product of the company. The data are collected by voluntary reporting from healthcare professionals when devices fail. As such, this leads to selection bias and underreporting of these adverse events. 

Despite these limitations, our study was able to analyze 93 reported complications spanning nine years. The results from this study may provide beneficial insights for operators on the most common failure modes and complications.

## Conclusions

Even though the RAPID registry has demonstrated the safety of the AngioVac system for suction thrombectomy, complications can still occur. The data from this paper serve to inform operators about potential risks and complications that may be associated with the device. It is imperative that physicians undergo adequate training to use the AngioVac system and understand the limitations of the device. Further studies are warranted to explore pulmonary embolism as a complication of AngioVac and its appropriate management.
